# Temporal and Spatial Characterization of Mononuclear Phagocytes in Circulating, Pulmonary Alveolar, and Interstitial Compartments in LPS-Induced Acute Lung Injury

**DOI:** 10.3389/fsurg.2022.837177

**Published:** 2022-03-02

**Authors:** Qi Li, Guoan Xiang, Shouchun Peng, Wenjie Ji

**Affiliations:** ^1^Department of Tuberculosis, Beijing Chest Hospital, Capital Medical University, Beijing, China; ^2^Department of Respiratory, The Third Medical Center of Chinese People's Liberation Army General Hospital, Beijing, China; ^3^Department of Respiratory, Affiliated Hospital of Armed Police Logistic College, Tianjin, China; ^4^Institute of Cardiovascular Disease and Heart Center, Pingjin Hospital, Tianjin, China

**Keywords:** acute lung injury, lipopolysaccharide, Ly6C, alveolar macrophages, interstitial macrophages

## Abstract

Peripheral circulating monocytes and resident macrophages are heterogeneous effector cells that play a critical role in the maintenance and restoration of pulmonary integrity. However, their detailed dynamic changes in lipopolysaccharide (LPS)-induced acute lung injury (ALI) remain unclear. Here, we investigated the impact of mononuclear phagocyte cells in the development of LPS-induced ALI/Acute respiratory distress syndrome (ARDS) and described the relations between the dynamic phenotypic changes and pulmonary pathological evolution. In this study, mice were divided into two groups and intraperitoneally injected with normal saline (NS) or LPS, respectively. A series of flow cytometry assay was performed for the quantification of peripheral circulating monocyte subpopulations, detection of the polarization state of bronchoalveolar lavage fluid (BALF)-isolated alveolar macrophages (AMϕ) and pulmonary interstitial macrophages (IMϕ) separated from lung tissues. Circulating Ly6C^lo^ monocytes expanded rapidly after the LPS challenge on day 1 and then decreased to day 7, while Ly6C^hi^ monocytes gradually increased and returned to normal level on the 7th day. Furthermore, the expansion of M2-like AMϕ (CD64^+^CD206^+^) was peaked on day 1 and remained high on the third day, while the polarization state of IMϕ (CD64^+^ CD11b^+^) was not influenced by the LPS challenge at all the time points. Taken together, our findings show that Ly6C^lo^ monocytes and M2-like AMϕ form the major peripheral circulation and pulmonary immune cell populations, respectively. The dynamic changes of mononuclear phagocyte in three compartments after the LPS challenge may provide novel protective strategies for mononuclear phagocytes.

## Introduction

Acute lung injury (ALI) is a pulmonary injury of alveolar epithelial cells and capillary endothelial cells, characterized by neutrophilic inflammation, diffuse pulmonary interstitial, and alveolar edema resulting in acute hypoxic respiratory insufficiency. Clinical manifestations of ALI include progressive hypoxemia, respiratory distress, and heterogeneous exudative lesions on lung imaging. ALI and its more severe stage acute respiratory distress syndrome (ARDS) occur in one-third of the patients with sepsis and have a high mortality rate of up to 40% (1, 2). However, there is no effective treatment for ALI, and the mechanisms of ALI/ARDS in sepsis remain unclear. Studies have shown that the main cause of ALI/ARDS is pulmonary vascular injury, which may lead to an increase in pulmonary vascular permeability (3–5). Lipopolysaccharide (LPS), a major component of the outer membrane of Gram-negative bacteria, is one of the most important pathogen-associated molecules of sepsis. LPS stimulation leads to a cascade of inflammatory responses resulting in lung injury. Therefore, inhibition of inflammatory cytokines is a potential treatment for ALI/ARDS.

The activation of innate and adaptive immunity leads to the aggravation of lung injury by releasing a large number of cytokines and inflammatory mediators. Monocytic phagocyte system cells are derived from immature myeloid cells and consist of a heterogeneous population of cells, providing circulating monocytes and resident recruited macrophages (Mϕ) that play an important role in innate pulmonary immune response (6, 7). Tam et al. reported that Ly6G–CD11b+Ly6Chi monocytes exhibited protective and immunosuppressive properties in inflammation (8). Our previous studies have revealed that there was a rapid expansion of circulating Ly6C^hi^ monocytes and M2-like AMϕ rather than the subsets of IMϕ in the bleomycin-induced pulmonary injury model (9). These results support the theory that there is an Ly6C^hi^-monocyte-directed pulmonary AMϕ alternative activation. Although studies have shown that subpopulations of mononuclear phagocytes have been recognized for their critical role in antimicrobial defense, the detailed temporal kinetic changes of mononuclear phagocytes in the circulating, pulmonary alveolar. Moreover, interstitial compartments in LPS-induced ALI/ARDS have not been described. In this study, we established an LPS-induced ALI mouse model and performed serial flow cytometry assays to investigate their association with the pathological evolution of pulmonary disease.

## Materials and Methods

### Reagents

Lipopolysaccharide (0111:B4 from *Escherichia coli*) was purchased from Sigma-Aldrich (St. Louis, MO, USA). 7-amino-actinomycin D (7-AAD) viability staining solution, PerCP/Cy5.5 anti-mouse Ly6G (clone 1A8), fluorescein isothiocyanate (FITC)-conjugated anti-mouse Ly6C (clone HK1.4), phycoerythrin (PE)-conjugated anti-mouse CD11b (clone M1/70), PE-conjugated anti-mouse CD206 (clone C068C2), PerCP/Cy5.5 anti-mouse CD64 (clone X54-5/7.1), and their respective isotype controls (PerCP/Cy5.5 Rat IgG2a, FITC Rat IgG2c, PE Rat IgG2b, PE Rat IgG2a, and PerCP/Cy5.5 Mouse IgG1) were purchased from Biolegend (San Diego, CA, USA).

### Animals

Male C57BL/6 mice at 8–10 weeks of age with body weights of 16–18 grams were purchased from the Laboratory Animal Center of the Academy of Military Medical Sciences (Beijing, China). All mice received human care in compliance with the Regulations for Management of Experimental Animals (Tianjin Municipal Science and Technology Commission, revised June 2004), which was in accordance with Guide for the Care and Use of Laboratory Animals published by the National Institutes of Health (NIH Pub. No.85-23, revised 1996). All procedures involving animals were approved by the Animal Use and Care Committee of the Pingjin Hospital.

### LPS-Induced ALI Mouse Model

In total, 60 mice were randomly divided into two groups: the LPS group (*n* = 30) and the normal saline (NS) group (*n* = 30). Mice were anesthetized by 2% isoflurane inhalation for 60–90 s followed by intraperitoneal (i.p.) injection of LPS (5 mg/kg) in 100 μl of sterile saline or an equal volume of NS. All mice had free access to food and water after the operation. On days 1, 3, and 7 after the LPS/saline challenge, mice were sacrificed under an overdose of pentobarbital sodium (100 mg/kg, *n* = 10 for each group at each time point). The blood samples were treated with ethylene diamine tetraacetic acid disodium salt (Na2-EDTA). The bronchoalveolar lavage fluid (BALF) and the lung tissues were collected for the following experiments.

### Histopathological Evaluation of the Lung Tissues

The non-lavaged lungs (left lobes) were fixed with 4% paraformaldehyde in phosphate buffer saline (PBS, pH 7.2–7.4) and inflated under pressure of −20 mmHg for 24 h. The paraformaldehyde-fixed left lobes were embedded in paraffin wax and sliced into 5 μm sections then stained with hematoxylin and eosin (H&E). To evaluate morphological characteristics, five successive selected areas of each lung section were observed under a magnification of ×200 using a light microscope (Olympus, Japan). The severity of alveolitis was determined according to the morphological characteristics, such as inflammatory infiltration and the lesion areas: score 0 represents the normal tissue; scores 1, 2, or 3 represent the degree of pulmonary inflammation <20, 20–50, or more than 50%, respectively. The average score of all examined areas was calculated as the inflammation score (IS).

### Bronchoalveolar Lavage Fluid Analysis

The BALF was harvested by lavage through the lung three times with 1 ml precooled saline each time *via* intratracheal cannula, and 90% of the total injected volume was consistently recovered. The BALF was centrifuged at 300 g for 15 min at 4°C. The cell-free supernatant was collected for the inflammatory cytokines assay, total protein quantification, and lactate dehydrogenase (LDH) detection (LDH Assay Kit, Jiancheng Bioengineering Institute, Nanjing, China). The cell pellets were resuspended in 0.9% sterile saline for total and differential cell counts and flow cytometry analysis.

### Flow Cytometry

Flow cytometry was performed to analyze the peripheral blood, lung cell suspensions, and BALF cell suspensions using a Cytomics FC500 Flow Cytometry (Beckman Coulter Inc., CA, USA) and FlowJo software (Treestar, Ashland, OR, USA) for data analysis. In monocytes phenotypic assays, hypotonic lysis was used to deplete the red blood cells prior to FCM detection. A volume of 30 μl Na2–EDTA anti-coagulated peripheral blood was stained for 15 min in the dark at room temperature using a mixture of anti-mouse antibodies specific for Ly6G (PerCP/Cy5.5), PE-conjugated CD11b, and FITC-conjugated Ly6C to identify Ly6C^hi^ and Ly6C^lo^ cells. In the AMϕ phenotypic assay, 50 μl BALF cell suspension was stained using a mixture of 7-AAD viability staining solution (eBioscience, Thermo Fisher Scientific, Inc., Waltham, MA, USA), anti-mouse antibodies specific for CD64 (PerCP/Cy5.5), PE-conjugated anti-mouse CD206, and PE-conjugated anti-mouse CD11b (BioLegend, CA, USA) according to the manufacturer's instructions to identify AMϕ M1 and AMϕ M2 cells. FCM assay was carried out after incubation. In IMϕ phenotypic assay, the right lower lung tissues were digested with 0.1% type I collagenase (Sigma-Aldrich, Inc., MA, USA) at 37°C for 1 h. After washing with D-Hanks and cell viability detection, 50 μl refiltered single-cell suspensions were stained in the presence of hypotonic lysis using a mixture of 7-AAD viability staining solution (eBioscience, Thermo Fisher Scientific, Inc., Waltham, MA, USA), anti-mouse antibodies specific for CD64 (PerCP/Cy5.5), PE-conjugated anti-mouse CD11b, and PE-conjugated anti-mouse CD206 (BioLegend, CA, USA) according to the manufacturer's instructions to identify IMϕ M1 and IMϕ M2 cells. FCM measurement was performed after incubation.

### Statistical Analysis

All data were expressed as means ± SEM from three replicate experiments and analyzed with one-way ANOVA using GraphPad Prism 5.0 software (GraphPad, San Diego, CA, USA) followed by the Bonferroni *post-hoc test* for statistical comparison of multiple groups. A two-tailed *p* < 0.05 was considered statistically significant.

## Results

### Lung Inflammatory Response in the LPS-Mediated ALI Model

**Figure 1:**
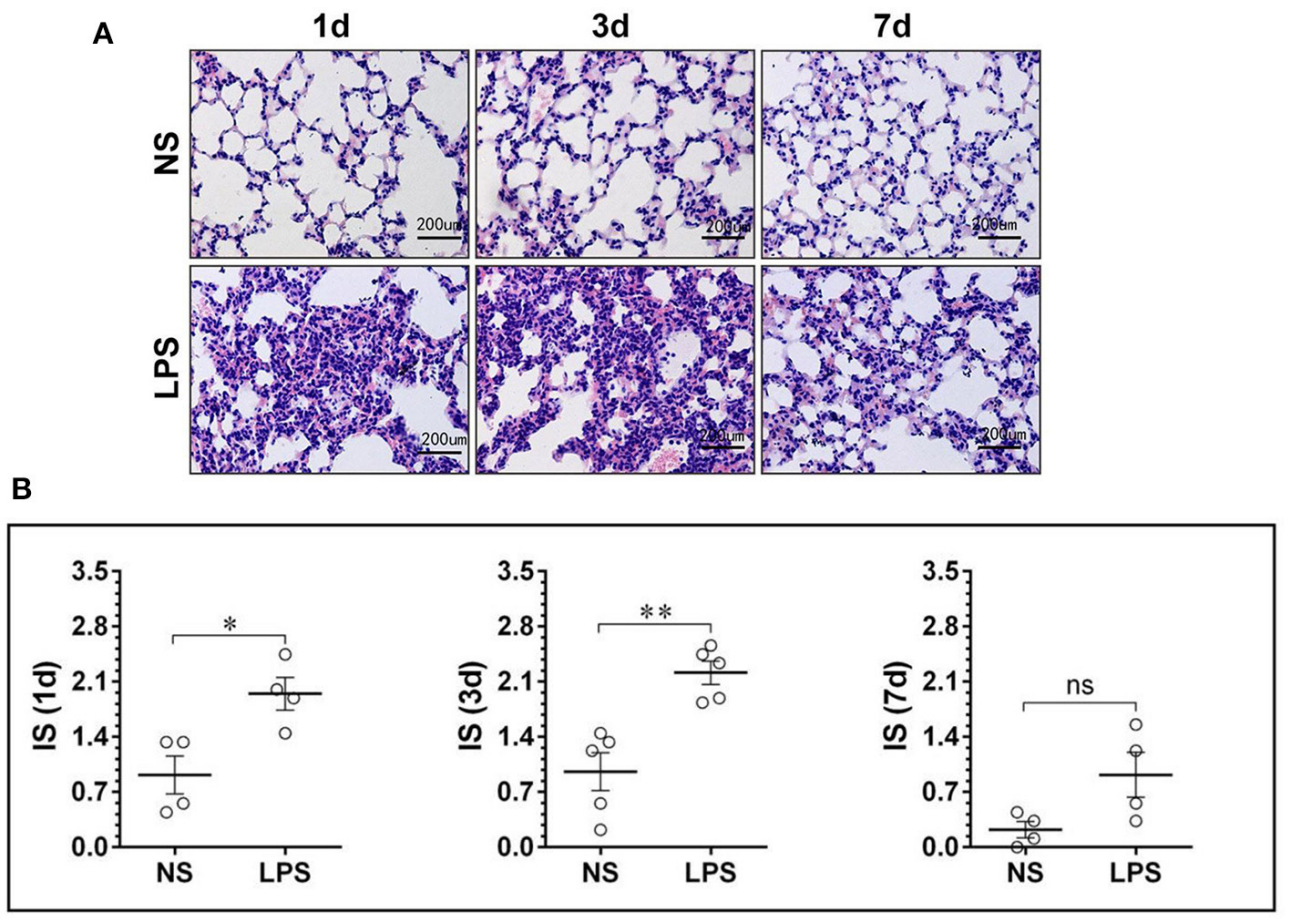
LPS-induced massive leukocyte infiltration and alveolar structural damage. **(A)** H&E staining lung tissue sections showed alveolar edema and swelled alveolar epithelium under stimulation of LPS. **(B)** The inflammation score of the NS and LPS groups at different time points. LPS, lipopolysaccharide; NS, normal saline; HE, hematoxylin and eosin; IS, inflammation score. Scale bars correspond to 50 μm in the micrographs. *p < 0.05, **p < 0.01. Statistical results were an average of five animals in each group.

**Figure 2:**
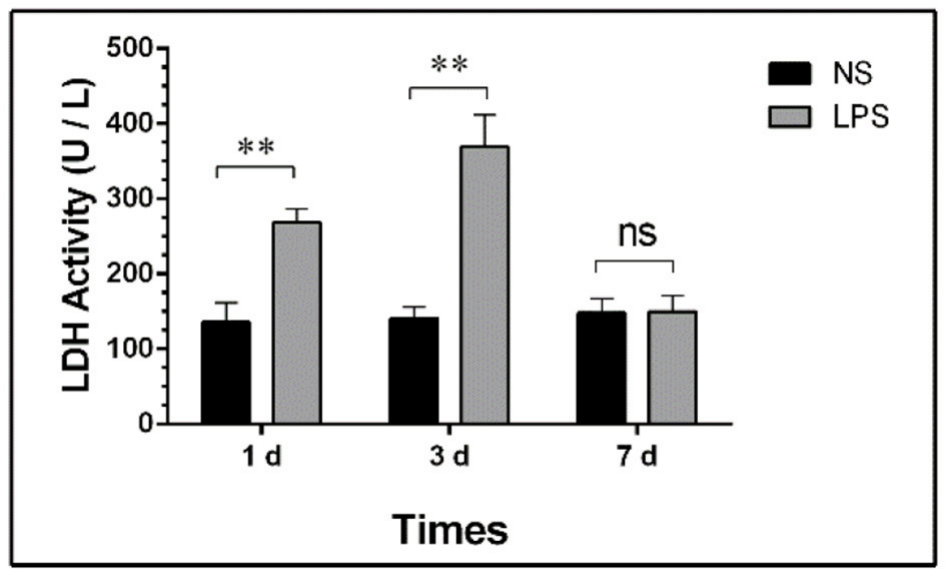
Total protein in BALF of LPS-induced ALI. Mice were sacrificed on days 1 and 3 after LPS stimulation and BALF were collected for protein quantification. BALF, bronchoalveolar lavage fluid; LPS, lipopolysaccharide; ALI: Acute lung injury; *p < 0.05; **p < 0.01; ns, no statistical significance. Statistical results were an average of five animals in each group.

To investigate the histological changes, the H&E-stained lung tissues were examined by light microscope on days 1, 3, and 7 after the LPS or NS challenge. As shown in Figures 1, 2, compared to the normal structure of lung tissue and pulmonary alveoli composed of thin septa, vascular, and connective tissue, LPS-induced ALI resulted in peribronchial and interstitial inflammatory cells significant infiltration with complications of acute congestion, edema, and alveolar wall thickening (Figure 1). Figure 2 shows the degree of inflammation in LPS groups is much higher than that in NS groups on days 1–3 and decreases to normal level on day 7.

### Total LDH Activity of BALF

**Figure 3:**
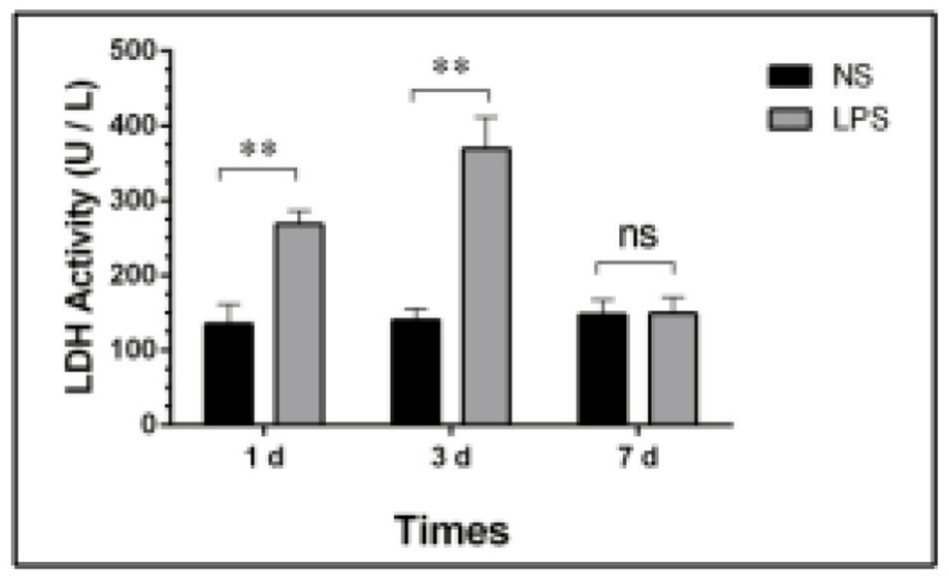
LDH activity in BALF of LPS-induced ALI. Mice were sacrificed on days 1, 3, and 7 after LPS stimulation and BALF were collected for LDH activity detection. BALF, bronchoalveolar lavage fluid; LPS, lipopolysaccharide; ALI, Acute lung injury; ** *p* < 0.01; ns, no statistical significance. Statistical results were an average of five animals in each group.

The concentration of total protein and LDH activity detection at each time point are shown in Figure 3.

### Temporal Dynamic Changes of Peripheral Circulating Monocyte Sub-Populations

**Figure 4:**
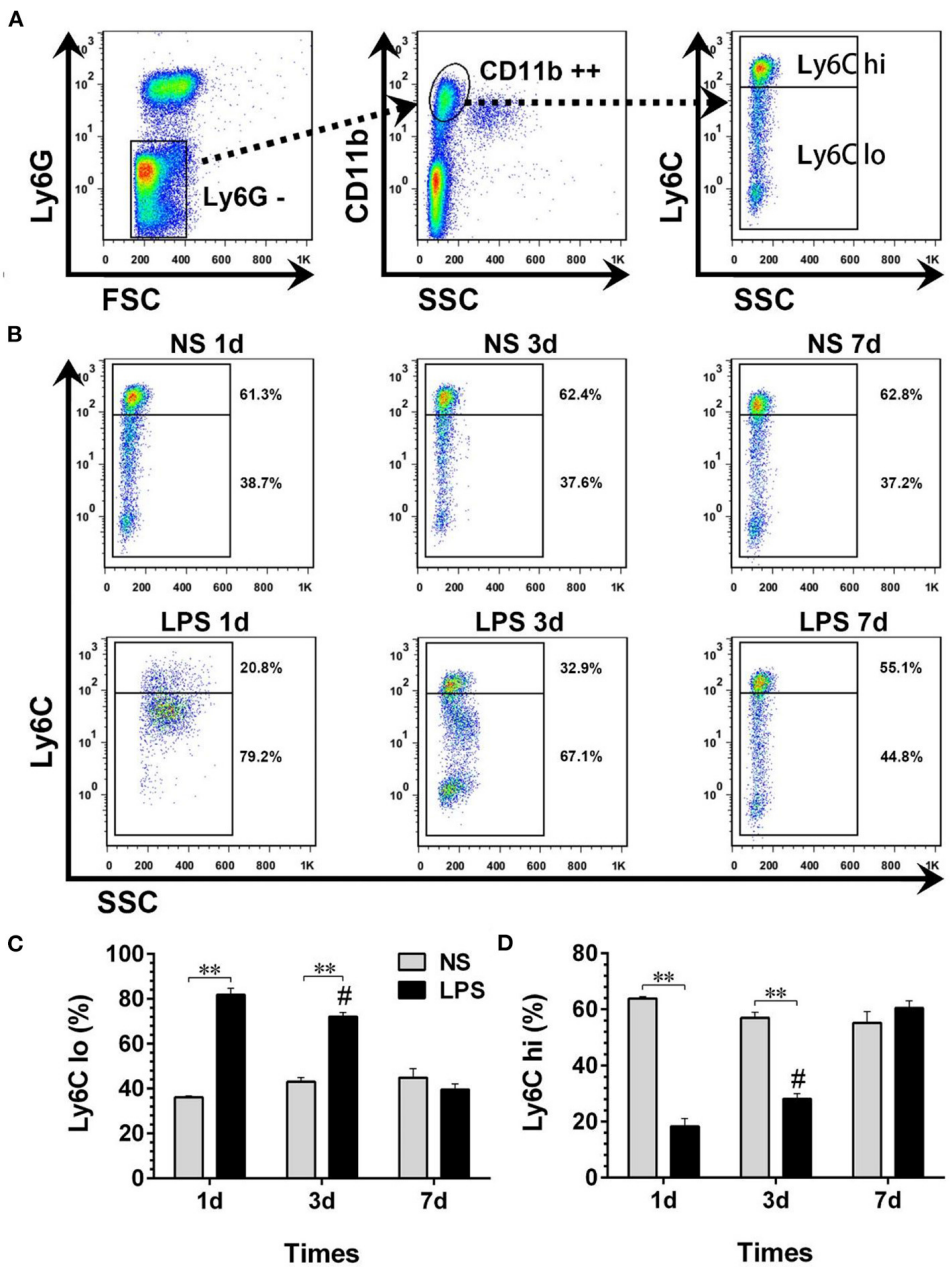
Temporal dynamic changes of peripheral circulating monocyte subpopulations after LPS stimulation. **(A, B)** Ly6C^hi^ and Ly6C^lo^ monocytes were identified by flow cytometry. Numbers outside boxed areas indicated the percentage of cells. The gating strategies for peripheral circulating monocyte subpopulations are shown in **(A)**. The dynamic characteristics of Ly6C^hi^ and Ly6C^lo^ monocytes in NS and LPS groups are shown in **(B)**. **(C, D)** Kinetics of increase in Ly6C^hi^ and Ly6C^lo^ monocyte numbers in peripheral blood. FSC, forward-scattered light; SSC, side-scattered light. #*p* < 0.05, compared with the LPS group on day 1; **p < 0.01. (*n* = 5 in each group).

The peripheral circulating monocytes presented a dynamic expression of Ly6C after LPS stimulation (Figures 4A,B). Compared with the NS group, the percentage of Ly6C^lo^ in the LPS group was highly increased on day 1 after the LPS challenge, then gradually declined from day 3 to day 7 (*p* < 0.05) and finally reached a level similar to that of the NS group (Figure 4C). In contrast with Ly6C^lo^, the temporal dynamics of Ly6C^hi^ remained at a lower level than that of the NS group until day 7 (Figure 4D).

### Temporal Dynamic Changes of BALF-Isolated AMϕ

**Figure 5:**
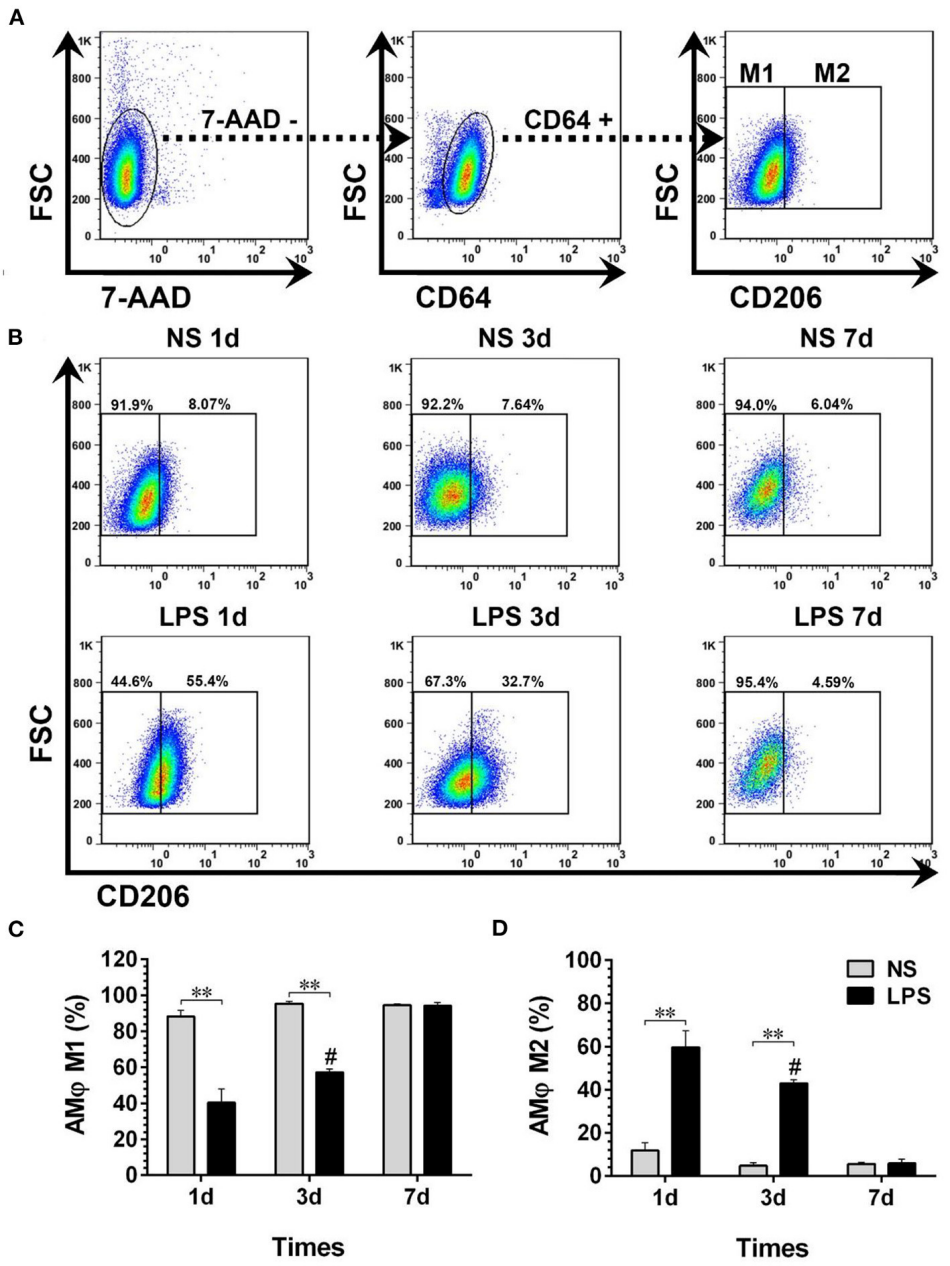
Temporal dynamic changes of AMϕ subpopulations after LPS stimulation. **(A, B)** M1- and M2-like AMϕ were identified by flow cytometry. Numbers outside boxed areas indicate the percentage of cells. The gating strategies for pulmonary AMϕ subpopulations is shown in **(A)**. The dynamic characteristics of lung M1- and M2-like AMϕ in NS and LPS groups are shown in **(B)**. **(C, D)** Kinetics of increase in M1- and M2-like AMϕ numbers in BALF-isolated cells. FSC, forward-scattered light; SSC, side-scattered light. #*p* < 0.05, compared with the LPS group on day 1; **p < 0.01. (*n* = 5 in each group).

The temporal dynamic changes of BALF-isolated AMϕ and IMϕ both in NS and LPS groups were characterized by FCM. Results showed that 7-AAD^−^CD64^+^CD206^−^ cells (M1-like AMϕ) accounted for the majority of cell types of BALF-isolated AMϕ in the NS group (Figures 5A,B). The percentage of M1-like AMϕ in the LPS group was low on day 1, then showed an upward trend from day 3 and finally reached a level similar to that of the NS group on day 7 (Figure 5C). However, the level of 7-AAD^−^CD64^+^CD206^+^ cells (M2-like AMϕ) in LPS treated mice was much higher than that in the NS group on day 1 then gradually decreased to normal level (Figure 5D).

### Temporal Dynamic Changes of BALF-Isolated IMϕ

**Figure 6:**
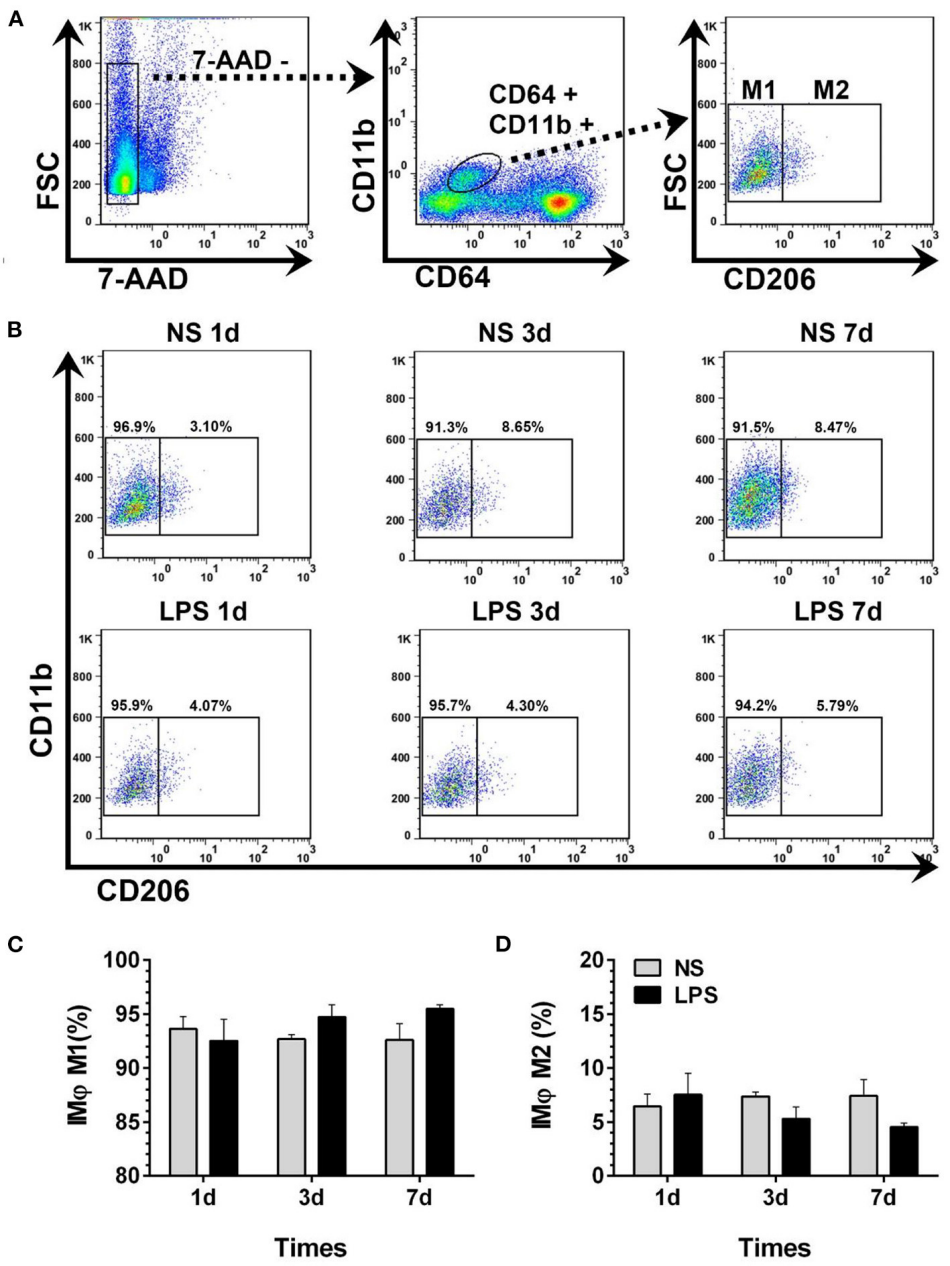
Temporal dynamic changes of IMϕ subpopulations after LPS stimulation. **(A, B)** M1- and M2-like IMϕ were identified by flow cytometry. Numbers outside boxed areas indicated the percentage of cells. The gating strategies for pulmonary IM subpopulations is shown in **(A)**. The dynamic characteristics of lung M1- and M2-like IMϕ in NS and LPS groups are shown in **(B)**. **(C, D)** Kinetics of increase in M1- and M2-like IMϕ numbers in the lung tissue. FSC, forward-scattered light; SSC, side-scattered light. #*p* < 0.05, compared with the LPS group on day 1; **p < 0.01. (*n* = 5 in each group).

Dynamic variations of IMϕ from digested lungs are shown in Figure 6. More than 92% of 7-AAD^−^CD64+CD11b^+^ cells showed M1-like phenotype (CD206–) (Figures 5A,B). Moreover, there was no significant change in M1 and M2 compared with normal levels from day 1 to 7 after LPS stimulation (Figures 6C,D).

## Discussion

Acute lung injury is a severe symptom of ARDS, characterized by capillary hyperpermeability, interstitial, and alveolar edema and aggregation of neutrophils, macrophages, and red blood cells in the alveoli. Although a large number of technical and supportive therapies have been developed by intensive care units over the past 40 years, few effective approaches have been used to treat ALI/ARDS, which has resulted in high mortality (2). LPS has a pro-inflammatory effect, triggering pulmonary inflammatory response through N oxide-dependent redox signaling (10) in pulmonary endothelial cells and causing alteration in the airway and pulmonary circulation function in mice (11, 12). i.p. injection of LPS has been widely acknowledged as a repeatable pharmacological model of ALI. Therefore, we used a mouse model of i.p. injection of LPS to observe the dynamics of three components of the mononuclear phagocyte system.

The present work manifested the temporal and spatial characterization of circulating monocytes, pulmonary resident macrophages, which include alveolar macrophage (AM) and interstitial macrophage (IM) in an ACI rodent model induced by i.p. injection of LPS. The main findings are all around these three monocyte-macrophage subpopulations. First, there was a higher level of Ly6C subset in the LPS group than that in the NS group on day 1 after LPS administration and declined subsequently and reached the same level as NS ultimately on day 7. Of note, there is a more active Ly6C^lo^ subset due to the LPS challenge, and by contrast, Ly6C^hi^ remained at a lower level than that of the NS group until day 7. Second, the temporal changes of M2-like AM are almost the same as Ly6C^lo^: an expansion of M2-like alveolar macrophage from day 1 and reached a similar level as that of the NS group on day 7. Third, with regard to IM, there was no significant difference between M1 and M2 compared with normal levels from day 1 to 7 after the LPS challenge. To the best of our knowledge, the present work is the first report with regard to describing the temporal and spatial changes of mononuclear phagocytes in the LPS-induced ACI model, which may provide clues for monocyte/macrophage targeting treatment of ACI or septic-ARDS. Moreover, we prove for the first time that LPS induces increased accumulation of M2-like AMϕ in the pulmonary alveoli rather than M1-like AMϕ.

As a component of the mononuclear phagocyte system, circulating peripheral blood monocytes provide a mobile and powerful cell source for innate immune system (13). They are also precursors of macrophages and dendritic cells and involved in the maintenance and restoration of tissue integrity (14, 15). It is noteworthy that in the previous studies of dynamics of circulating monocytes, AMϕ and IMϕ in BLM-induced lung injury and fibrosis mouse model, mouse Ly6C^hi^ monocyte was a therapeutic target for many inflammatory diseases, which supported the Ly6C^hi^ directed pulmonary alternative activation mechanism (16).

Compared to active Ly6C^hi^ in fibrosis mice, the Ly6C^lo^ subpopulation was more active after LPS exposure. The mechanism behind this phenomenon may be that when the cells are damaged, the Ly6C^lo^ subpopulation with more surface CX3CR1 expression, which can crawl or patrol in the vascular cavity faster, and can be seen as sentinel cells (27), and neutrophils can migrate to the injured site by Ly6C^lo^ cells secreting CXCL2 chemokine (28). Moreover, it is also reported that the accumulation of Ly6C^low^ monocytes was associated with the expression of adhesion molecules in vascular endothelial cells (17), and LPS-induced tumor necrosis factor α (TNF-α) release resulted in intercellular adhesion molecule (ICAM-1) and E-selectin expression as well as increased vascular cell adhesion molecule (VCAM-1) expression in supernatant-activated endothelial cells. Therefore, LPS is a trigger for the indirect activation of endothelial cells by monocytes, which plays an important role in the adhesion of breast cancer cells (18). According to these findings, we propose that LPS-induced adhesion molecules expression contributes to the accumulation of Ly6Clow monocytes in the peripheral circulation and local infiltrate of M2-like AMϕ in the pulmonary alveoli. Additionally, it is reported that selective depletion of Ly6C^lo^ subpopulation would lengthen the duration of inflammation absorption and regression (19), and furthermore, Shichino et al. (20) believed that Ly6C^lo^ restorative macrophage subset plays a role of candidate lung macrophages and could different into the monocyte-derived macrophages (MMs) in alveoli as a result of Ly6C^lo^ subset cells and MMs response to inflammation both in a C-C chemokine receptor type 2 (CCR2)-dependent manner (21). Although we observed the ACI in mice within 7 days, according to the comparison betweensilica-induced pulmonary fibrosis mice and LPS-induced ARDS or ALI mice, we can reasonably speculate that LPS can cause a slight rise of M2 phenotype alveolar macrophages for a short time, while it would cause secondary fibrosis change in the lung tissue later as well. The magnitude of the LPS-induced lung lesion should be less than the typical pulmonary fibrosis caused by bleomycin and silica. We believe that there is a positive correlation between M2 phenotype alveolar macrophages proportion in the early stage with the degree of prognosis in the later stage, and it also has a certain suggestive effect on the degree of lung injury at that time.

In conclusion, this study demonstrates the dynamic changes of circulating monocytes, AMϕ and IMϕ, in the LPS-induced ALI mouse model. A rapid expansion of circulating Ly6C^lo^ monocytes and M2-like AMϕ suggests that monocyte phagocytes may be a potential therapeutic target for ALI (22).

## Data Availability Statement

The original contributions presented in the study are included in the article/supplementary material, further inquiries can be directed to the corresponding author.

## Ethics Statement

The animal study was reviewed and approved by Animal Use and Care Committee of the Beijing Chest Hospital, Capital Medical University.

## Author Contributions

QL is the mainly responsible for the writing of the article. GX is mainly responsible for research design. SP is mainly responsible for data analysis. WJ is responsible for the guidance of the entire research. All authors listed have made a substantial, direct, and intellectual contribution to the work and approved it for publication.

## Conflict of Interest

The authors declare that the research was conducted in the absence of any commercial or financial relationships that could be construed as a potential conflict of interest.

## Publisher's Note

All claims expressed in this article are solely those of the authors and do not necessarily represent those of their affiliated organizations, or those of the publisher, the editors and the reviewers. Any product that may be evaluated in this article, or claim that may be made by its manufacturer, is not guaranteed or endorsed by the publisher.
